# Extensive Type V Truncal Aplasia Cutis Congenita in a Surviving Twin With *Fetus Papyraceus*: A Case Report

**DOI:** 10.1155/crpe/4089919

**Published:** 2026-02-02

**Authors:** Assaf Ghallab Alharbi, Khalid Nabil Nagshabandi, Hala Abdullah Almusa, Bandar AlHarbi, Abdullah Surrati, Bushra Alradadi

**Affiliations:** ^1^ Department of Dermatology, Ohud Hospital, Medina, Saudi Arabia, ohudhospital.com; ^2^ Department of Dermatology, College of Medicine, King Saud University, Riyadh, Saudi Arabia, ksu.edu.sa; ^3^ Department of Dermatology and Allergy, Clinical Research Center for Hair and Skin Science, Charité-Universitätsmedizin, Berlin, Germany, charite.de; ^4^ College of Medicine, King Saud University, Riyadh, Saudi Arabia, ksu.edu.sa

**Keywords:** aplasia cutis congenita, calcipotriol, *fetus papyraceus*, ischemic skin necrosis, neonatal dermatology, type V ACC, vanishing twin syndrome, vascular disruption

## Abstract

**Background:**

Aplasia cutis congenita (ACC) is a rare developmental anomaly characterized by the absence of skin at birth. Type V ACC is a distinct subtype associated with *fetus papyraceus*—the compressed remnant of a deceased co‐twin—and typically manifests as symmetrical, linear, or stellate lesions involving the trunk and proximal extremities. The proposed mechanism involves ischemic or thromboembolic injury to the surviving twin due to shared placental circulation.

**Case Presentation:**

We describe a 30‐day‐old female neonate, the surviving twin of a dichorionic gestation, whose co‐twin was delivered as a *fetus papyraceus*. The patient presented with extensive, bilaterally symmetrical, linear, and plaque‐like areas of absent skin involving the anterior and posterior trunk. No scalp, limb, or mucosal involvement was observed. Systemic examination and laboratory workup were unremarkable. Based on the characteristic lesion pattern and obstetric history, a diagnosis of type V ACC was established. The patient was treated conservatively with topical calcipotriol and meticulous wound care, leading to progressive re‐epithelialization and residual atrophic scarring.

**Conclusion:**

This case illustrates the classic presentation of type V ACC associated with *fetus papyraceus* and supports the vascular disruption hypothesis as the predominant pathogenic mechanism. Awareness of this rare entity facilitates early diagnosis, appropriate conservative management, and accurate counseling of parents regarding the excellent prognosis and low recurrence risk.

## 1. Introduction

Aplasia cutis congenita (ACC) associated with fetus papyraceus represents a distinct clinicopathologic entity within the spectrum of congenital skin defects. ACC is characterized by localized or extensive absence of skin present at birth, most commonly affecting the scalp, but in this subtype, lesions typically involve the trunk, buttocks, and proximal limbs in a bilateral and symmetric distribution [1–3]. The pathogenesis is closely linked to the intrauterine demise of a co‐twin during the late first or early second trimester, resulting in the so‐called fetus papyraceus—an atrophic, compressed fetal remnant. The prevailing hypothesis implicates vascular disruption or ischemic insult to the surviving twin, likely mediated by shared placental circulation, as the mechanism underlying the cutaneous defects [1, 4–6].

This association is classified as type V ACC in the Frieden system and is notable for its reproducible lesion pattern and potential for additional anomalies, such as pulmonary involvement [2, 5, 7]. Although rare, with fewer than 50 cases reported in the literature, recognition of this entity is critical for appropriate diagnosis, management, and counseling regarding prognosis and recurrence risk [3, 5, 8]. Conservative wound care remains the mainstay of treatment, with most cases demonstrating favorable healing outcomes [2, 5, 9].

## 2. Case Presentation

A 30‐day‐old Saudi Arabian (Arab) female neonate was referred to the dermatology clinic for evaluation of multiple skin defects present since birth. She was born full term via cesarean section due to breech presentation to a 38‐year‐old healthy multiparous mother (G8P7 + 1). The pregnancy was uneventful until delivery. Routine antenatal obstetric ultrasonography confirmed the twin gestation and subsequently documented intrauterine demise of the co‐twin at approximately 20 weeks of gestation, consistent with fetus papyraceus; no structural anomalies were reported in the surviving twin. The infant was the second of a twin gestation; however, her co‐twin, a male fetus, had died in utero and was delivered as a fetus papyraceus. There was no history of maternal illness, teratogenic drug exposure, or intrauterine infection. At birth, the baby was noted to have extensive areas of absent skin over the trunk and abdomen. She was admitted to the neonatal intensive care unit (NICU) for 7 days for management of the skin lesions and mild respiratory distress. Initial laboratory investigations, including a complete blood count, were within normal limits, and sepsis was ruled out. Her blood group was B positive, and the direct Coombs test was negative. She passed urine adequately, and no metabolic or systemic abnormalities were detected.

**Figure 1:**
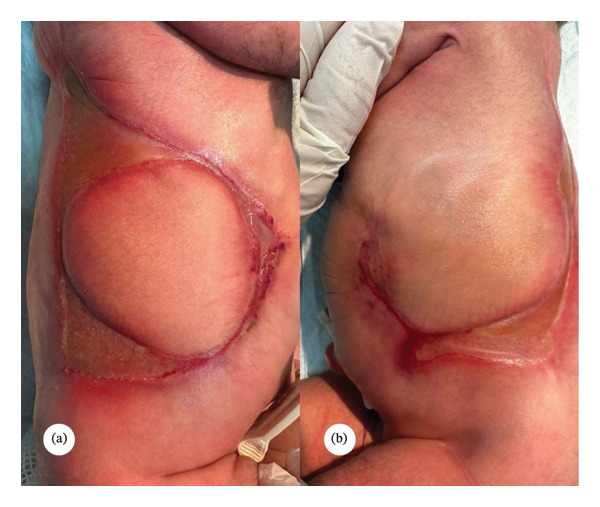
(a), (b) Bilaterally symmetrical, well‐demarcated linear and plaque‐like areas of absent skin over the trunk in a neonate, partially re‐epithelialized with thin atrophic membranes and surrounded by erythematous borders.

**Figure 2:**
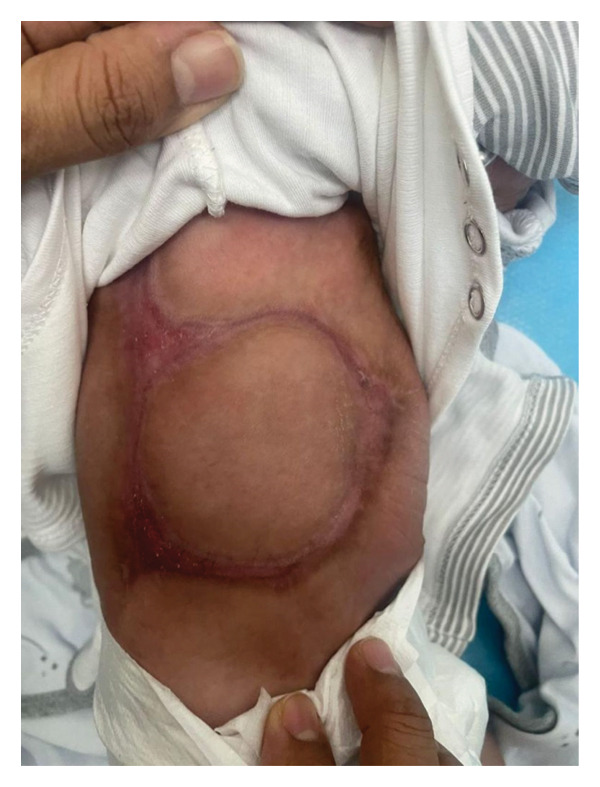
Follow‐up photograph showing significant re‐epithelialization of the previously denuded truncal area after 4 months of conservative management with topical calcipotriol and gentle wound care. The lesion has healed with a well‐defined, smooth, atrophic scar surrounded by a faint erythematous rim, and no signs of secondary infection are present.

On examination at 1 month of age, the infant appeared stable, active, and not in distress. Cutaneous examination revealed well‐demarcated linear streaks and plaque‐like areas of absent skin involving the anterior and posterior trunk, extending to the abdomen (Figure 1). No scalp, limb, or mucosal involvement was seen. The rest of the physical examination was unremarkable. Because of her breech presentation at birth, a screening ultrasound of the hips was performed to rule out developmental dysplasia. The study demonstrated normal alpha and beta angles bilaterally (*α*: 62° on the right, 63° on the left; β: 48° on the right, 46° on the left), confirming normal hip development. Given the characteristic distribution of lesions, the absence of systemic involvement, and the history of a co‐twin demise, a clinical diagnosis of extensive type V ACC associated with fetus papyraceus was made. Amniotic band syndrome and traumatic ischemic necrosis were considered less likely and were ruled out. The patient was started on topical calcipotriol ointment once daily to promote epithelialization and was advised on meticulous local wound care to prevent secondary infection. The parents were counseled about the benign nature of the condition and the likelihood of residual scarring. At the 4‐month follow‐up, the lesions showed complete epithelialization with atrophic scarring and were surrounded by hyperpigmented borders, with no evidence of secondary infection (Figure 2). At the family’s request, a referral to a tertiary dermatology center was arranged for further evaluation and long‐term follow‐up.

## 3. Discussion

ACC encompasses a heterogeneous group of congenital disorders characterized by the absence of localized or extensive areas of skin, with or without involvement of deeper tissues [1]. While the scalp remains the most common site, extracranial lesions—particularly those involving the trunk and limbs—are often associated with specific underlying etiologies. Type V ACC, as defined by Frieden, is associated with fetus papyraceus or vanishing twin syndrome and is characterized by extensive, bilaterally symmetrical, linear or stellate lesions predominantly involving the trunk and proximal extremities [2, 3, 5].

Our case exhibited the hallmark features of type V ACC: symmetric, truncal, plaque‐like areas of skin absence in a surviving twin following the intrauterine demise of the co‐twin. Similar presentations have been documented by Leung et al. and Qureshi et al., who reported nearly identical linear or circumferential truncal lesions in monochorionic twin pregnancies complicated by fetal demise [2, 8]. The predominant pathogenic mechanism is believed to be vascular compromise, resulting from embolization, thrombosis, or ischemic hypotension following co‐twin death [1, 4–6]. The symmetric distribution of lesions along so‐called “watershed zones” supports this ischemic etiology [6]. Histopathological examinations in previous reports revealed the absence of dermis and epidermis consistent with ischemic necrosis rather than developmental failure [4, 6]. Cambiaghi et al. further reinforced this theory by identifying hepatic hematomas and visceral ischemic changes in surviving twins, suggesting a systemic hypovolemic‐ischemic event secondary to placental vascular disruption [6]. Morrow et al. proposed that transient hypotensive episodes during feto‐fetal transfusion could explain the bilateral truncal lesion pattern [7]. In addition to the vascular theory, some authors have postulated mechanical compression or amniotic adhesion as contributing factors, though these are less consistent with the symmetrical distribution typically seen in type V ACC [5].

Prenatal evaluation may sometimes suggest the diagnosis. Routine obstetric ultrasonography can identify the intrauterine demise of a co‐twin and the compressed fetal remnant (fetus papyraceus). Elevated maternal serum alpha‐fetoprotein (AFP), positive acetylcholinesterase bands in amniotic fluid, and a small fetal abdominal circumference have been described in pregnancies complicated by fetus papyraceus and later confirmed cases of ACC [9, 10]. However, these findings are not specific, and diagnosis is usually established postnatally based on the combination of lesion morphology and obstetric history [3, 5, 8]. To date, maternal multiparity has not been recognized as an independent risk factor for fetus papyraceus or type V ACC; rather, risk is primarily driven by multiple gestation and placental vascular/hemodynamic events after co‐twin demise [3, 5]. Our patient was managed conservatively with topical calcipotriol and meticulous wound care, achieving progressive re‐epithelialization and eventual scarring—similar to the favorable outcomes reported in conservatively treated cases [2, 9]. For extensive or deep lesions, surgical intervention may be required, with autologous grafts or dermal regeneration templates such as Integra showing positive outcomes in selected cases [11]. Overall, this case underscores the importance of recognizing the characteristic distribution and obstetric context of type V ACC. Differentiating it from amniotic band syndrome, trauma, or congenital infections prevents unnecessary investigations and enables accurate counseling regarding its benign course and low recurrence risk. A limitation of this report is that only lateral‐view clinical photographs were available, and additional anterior/posterior images could not be obtained.

## 4. Conclusion

ACC associated with *fetus papyraceus* is a distinct form of congenital skin absence resulting from vascular or ischemic injury after co‐twin demise. Diagnosis is clinical, supported by the characteristic symmetrical truncal lesions. Conservative management yields excellent outcomes with complete epithelialization and minimal scarring. Early recognition and parental counseling are essential for optimal care and differentiation from other congenital skin defects.

## Funding

No funding was received for this manuscript.

## Ethics Statement

Ethical approval is not required for this study in accordance with local or national guidelines.

## Consent

Written informed consent was obtained from the patient for publication of the details of their medical case and any accompanying images.

## Conflicts of Interest

The authors declare no conflicts of interest.

## Data Availability

The data that support the findings of this study are available from the corresponding author upon reasonable request.
